# Case of Successful Immediate Suturing of the Olecranon for Simple Fracture

**Published:** 1884-03

**Authors:** W. P. Keall

**Affiliations:** Surgeon to the Bristol General Hospital


### CASE OF SUCCESSFUL IMMEDIATE SUTURING OF THE OLECRANON FOR SIMPLE FRACTURE. By W. P. Keall, M.R.C.S., Surgeon to the Bristol General Hospital.

Samuel Taylor, æt. 33, Cab-driver, was admitted into the Bristol General Hospital, November 28th, 1883, with a simple fracture of the olecranon, caused by a blow with a heavy iron bar, inflicted on the arm, which was raised to protect his head. The fragments were separated more than an inch when the arm was straightened. He was suffering severe pain, there was some swelling and a good deal of deformity about the elbow, but very little ecchymosis, one small spot of skin, however, about 2 mm. in size was severely bruised.

November 29th, the operation was performed in the following manner: An incision about four inches long was made from the tip of the olecranon downwards, over the posterior aspect of the ulna, similar to the lower part of the incision for excision of the elbow-joint. The soft parts were cleared away for a small space from the posterior surface of the fragment, and a hole drilled through to the broken surface, avoiding the articular cartilage; through this a stout silver wire was passed from the outside, inwards. The tissues were then in a like manner separated from the ulna, and another hole drilled in the same way, except that from the angle made by the two surfaces. The direction of this one had to be made more obliquely than the first; and in this I was more fortunate than Sir Joseph Lister was in the case he describes, and of which he gives a wood-cut in the *British Medical Journal* for November 3rd, 1883, therefore there was no need for me to adopt the very ingenious manœuvre carried out by him with the needle and gouge.

The joint, of which the articular cartilage, especially that covering the surface of the humerus, was plainly visible, was cleared of blood clot and *débris* from the drilling the bone; another wire was passed through this from without inwards, and the end of it between the two fragments of bone was passed through a loop formed at the internal end of the first wire, by which it was pulled out through the first hole. The two pieces of bone then being well pressed together, the two ends of the wire were drawn tight, twisted together, cut off, bent and hammered down on to and partially into the bone. A drainage tube having been inserted, the wound was stitched up and dressed with the protective and gauze in the usual manner.

The same afternoon, at 4.30, some two or three hours afterwards, he said he was in a great deal of pain, but not so much as before the operation. Temperature rose that evening to 99°.4, pulse 84.

November 30th, the next day, the wound on being dressed looked very well; there was only a small amount of discharge. The drainage tube was shortened; he felt fairly comfortable; considerably less pain than before the operation.

Temperature in the morning, 99°; evening, 99°.8, being the highest point reached, pulse 76.

December 1st.—Dressed wound, which looked remarkably well; there was a discharge of about three drachms of serum; drainage tube shortened again. Slept well, took his food well, and felt comfortable.

December 2nd.—Dressed wound, discharge about one drachm serum; all drainage removed; slight passive movement of the joint made on that day; temperature normal.

December 4th.—Wound dressed; all united, except at the part where the drainage tube had been; removed the stitches; very little discharge.

December 5th.—The wound was again dressed to-day, because he said he felt as if the discharge were running down his arm, which was hanging down, being on a straight splint; but on removing the dressings there was scarcely any discharge, the skin was well united, and the drainage tube cavity filled up. There was some slight tenderness behind the internal condyle, but this was relieved on altering the dressing, and was, I believe, caused by the bandage having been rather tight above the elbow.

From this time he went on steadily improving, passive movements being made frequently. The arm was put on an outside iron splint, hinged at the elbow, which he was allowed to remove and bandage on again after the first ten days.

At the end of the fourth week he could bend the joint to a right angle; at six weeks he was shown at the January meeting of the Bristol Medico-Chirurgical Society, when it was seen that most of the movements of the elbow were almost perfect. He could extend it until almost straight, bend it to less than a right angle, could touch his head and could pronate and supinate, and seven weeks after the operation he resumed his work as a cab-driver.

I do not for a moment mean to advocate this heroic treatment in every case of fractured olecranon, for the last which came under my care made a very good recovery with bony union, but in that the fragment was smaller, and was easily brought into perfect apposition with the rest of the ulna. The arm, of course being extended, was put up in a gum bandage, which was rendered perfectly rigid by long strips of tin, about $\frac{3}{4}$in. wide, being placed between the layers of the bandage. Nevertheless, he had not all the movements of his arm restored in six weeks. However, I do feel pretty strongly that if this method is to be adopted at all, it is very much better to do it as soon as possible after the accident, and not to wait until the fragment has been partly absorbed, when, of course, the two surfaces will not correspond; and, in fact, it may be necessary to remove still more of the bone in order to get an adhesive surface, to say nothing of the obstructions to the restoration of the joint by the possible organisation of blood clot, &c., within it, together with any adhesions that may have taken place.

Although primary wiring of the olecranon has never yet been advocated, it was so evident in this case that bony union was almost if not quite impossible that I determined to wire it at once, and think the result has quite borne out the propriety of that determination.

Of course, I should not feel justified in risking such a grave condition as a compound fracture into an important joint if I were not certain that the most perfect antiseptic precautions would be taken; but my mind was rendered perfectly easy on that score, and I feel deeply indebted to our house surgeon, Mr. Penny, for the very careful and successful way in which he superintended the dressings of this as well as many more of my cases after operation.

The temperature, as shown by the chart, is typical of an aseptic operation. There was slight elevation on the day of and the one following the operation, rising to a max. of about 100 Fah., in this case 99°.8, which is probably the result of nervous disturbance. This quickly subsided to a normal, or taking 98°.4 as the normal to a slightly sub-normal temperature.

In order to avoid any chance of splitting the bone, I used an ordinary Archimedean drill to bore the holes, but as it is difficult to fix the fragment while both hands are employed in working the drill, I think an ordinary bradawl, as used by Professor Lister, would be a better instrument for that purpose.

In the next case I should instead of the first wire pass a loop of rather finer wire, and through it pass the end of the second wire, because in making the loop at the end of the first, on retracting it there was a tendency for the turned-up end to hitch.

